# Differential Gene Expression Analysis of Bovine Macrophages after Exposure to the *Penicillium* Mycotoxins Citrinin and/or Ochratoxin A

**DOI:** 10.3390/toxins9110366

**Published:** 2017-11-13

**Authors:** Kristen M. Brennan, Se-Young Oh, Alexandros Yiannikouris, Daniel E. Graugnard, Niel A. Karrow

**Affiliations:** 1 Center for Animal Nutrigenomics and Applied Animal Nutrition, Alltech Inc., Nicholasville, KY 40356, USA; ayiannikouris@alltech.com (A.Y.); dgraugnard@alltech.com (D.E.G.); 2 Department of Animal Biosciences, University of Guelph, Guelph, ON N1G2W1, Canada; ohs@uguelph.ca (S.-Y.O.); nkarrow@uoguelph.ca (N.A.K.)

**Keywords:** ochratoxinA, citrinin, mixture toxicity, bovine macrophage, gene expression, microarray

## Abstract

Mycotoxins produced by fungal species commonly contaminate livestock feedstuffs, jeopardizing their health and diminishing production. Citrinin (CIT) and ochratoxin A (OTA) are mycotoxins produced by *Penicillium* spp. and commonly co-occur. Both CIT and OTA can modulate immune response by inhibiting cell proliferation and differentiation, altering cell metabolism, and triggering programmed cell death. The objective of this study was to determine the effects of sublethal exposure (i.e., the concentration that inhibited cell proliferation by 25% (IC_25_)) to CIT, OTA or CIT + OTA on the bovine macrophage transcriptome. Gene expression was determined using the Affymetrix Bovine Genome Array. After 6 h of exposure to CIT, OTA or CIT + OTA, the number of differentially expressed genes (DEG), respectively, was as follows: 1471 genes (822 up-regulated, 649 down-regulated), 5094 genes (2611 up-regulated, 2483 down-regulated) and 7624 genes (3984 up-regulated, 3640 down-regulated). Of these, 179 genes (88 up-regulated, 91 down-regulated) were commonly expressed between treatments. After 24 h of exposure to CIT, OTA or CIT + OTA the number of DEG, respectively, was as follows: 3230 genes (1631 up-regulated, 1599 down-regulated), 8558 genes (4167 up-regulated, 4391 down-regulated), and 10,927 genes (6284 up-regulated, 4643 down-regulated). Of these, 770 genes (247 up-regulated, 523 down-regulated) were commonly expressed between treatments. The categorization of common biological functions and pathway analysis suggests that the IC_25_ of both CIT and OTA, or their combination, induces cellular oxidative stress, a slowing of cell cycle progression, and apoptosis. Collectively, these effects contribute to inhibiting bovine macrophage proliferation.

## 1. Introduction

Mycotoxins are the secondary metabolites of fungal species. Their ubiquitous presence in plant commodities makes contamination of the feed supply chain virtually unavoidable and problematic [1]. Acute or chronic exposure of livestock to mycotoxins can increase morbidity and decrease production by adversely affecting reproduction, feed efficiency and growth [2,3]. 

Citrinin (CIT) and ochratoxin A (OTA), mycotoxins produced by *Aspergillus* spp. and co-produced by *Penicillium* spp., commonly occur together in feedstuffs such as cereals and forages. Previous research has shown that cellular damage and immunosuppression are associated with *Penicillium* mycotoxin (PM) exposure. Ochratoxin A primarily targets the kidneys, but is also immunomodulatory, hepatotoxic, genotoxic, and carcinogenic in other organs such as the brain and intestine [4,5,6,7]. Studies suggest that OTA is a potent inhibitor of the renal phosphoenolpyruvate carboxykinase, thus impacting gluconeogenesis, and also interrupts normal cell function by inhibiting mitochondrial energy production and respiration, increasing lipid peroxidation, and altering protein synthesis by disrupting phenylalanine metabolism [8,9,10]. Citrinin is also nephrotoxic and genotoxic [11] and alters the mitochondrial activity and homeostasis of reactive oxygen species (ROS) [12]. Like OTA, CIT is also immunomodulatory [13], and exposure to the combination of CIT and OTA has been shown to result in additive and synergistic effects on cellular toxicity [14,15,16]. Most *Penicillium* spp. are found in combinations in nature, so animals are seldom exposed to isolated mycotoxins alone [17]. 

Immunomodulation by CIT and OTA can occur by the inhibition of cell proliferation and differentiation, by changes in cell metabolism, and by triggering apoptosis [18,19,20,21]. Oh et al. showed that CIT, OTA and the combination of CIT + OTA can inhibit macrophage proliferation, with the most potent toxic effects resulting from their combination [16]. Exposure to PM can also change the epigenome resulting in the suppression of RNA synthesis, potentially indirectly modulating normal immune cell functions like proliferation [22,23,24]. While studies have shown that both CIT and OTA can inhibit immune cell metabolism and trigger apoptosis, there is little information on the molecular mechanisms behind these immunomodulatory effects. The objective of this study was to determine the effects of sublethal exposure (i.e., the concentration that inhibited cell proliferation by 25% (IC_25_)) to CIT, OTA or the combination of CIT + OTA on the bovine macrophage (BoMac) transcriptome. 

## 2. Results and Discussion

### 2.1. Summary of Differentially-Expressed Genes Identified from Microarray Analysis

**Figure 1:**
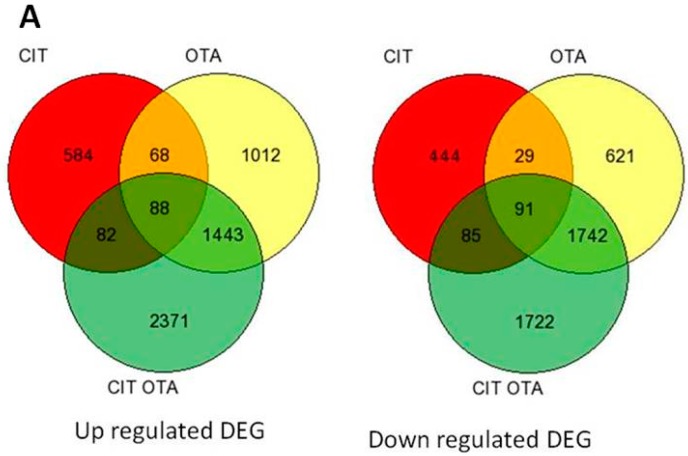

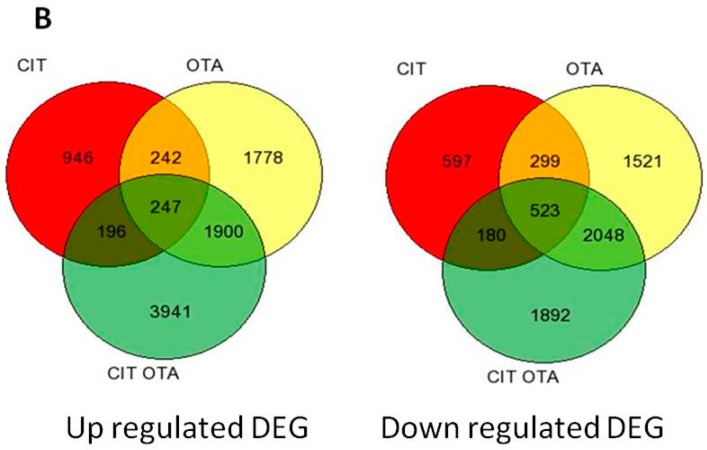
Common and unique differentially expressed genes in bovine macrophages after exposure to CIT, OTA, or CIT + OTA for (**A**) 6 h and (**B**) 24 h.

Interpretation of the genome-wide transcriptional changes indicates that both alone and in combination, CIT and OTA modulate bovine macrophage cell function in part by increasing cellular stress and decreasing cell cycle progression. From the total of 24,341 genes on the microarray, the numbers of differentially expressed genes (DEG), relative to the ethanol control for each time point, are shown in Figure 1. After 6 h of exposure to CIT, OTA or CIT + OTA the number of DEG, respectively, were as follows (Table S1): 1471 genes (822 up-regulated, 649 down-regulated), 5094 genes (2611 up-regulated, 2483 down-regulated) and 7624 genes (3984 up-regulated, 3640 down-regulated). Of these, 179 genes (88 up-regulated, 91 down-regulated) were commonly expressed between treatments. After 24 h of exposure to CIT, OTA or CIT + OTA the number of DEG, respectively, were as follows (Table S2): 3230 genes (1631 up-regulated, 1599 down-regulated), 8558 genes (4167 up-regulated, 4391 down-regulated) and 10,927 genes (6284 up-regulated, 4643 down-regulated). Of these, 770 genes (247 up-regulated, 523 down-regulated) were commonly expressed between treatments. Real-time PCR validation confirmed changes in select DEG (Table S3). 

### 2.2. Transcriptional Changes after 6 h of Exposure to PM

The most up-regulated DEGs in the CIT treatment group were sulfiredoxin 1 (SRXN1, 5.04 fold) and oxidative stress induced growth inhibitor 1 (OSGIN1, 3.41 fold). Sulfiredoxin codes for a protein that is critical for maintaining redox balance and is triggered to help protect cells from oxidative stress-induced apoptosis [25], whereas OSGIN1 is an oxidative stress-responsive protein that regulates apoptotic cell death [26]. The most frequently down-regulated DEG in the CIT treatment group included 2′-5′-oligoadenylate synthetase 1 (OAS1, −2.22 fold) and GDP-mannose pyrophosphorylase B (GMPPB, −1.820 fold). While the exact role of GMPPB during mycotoxin challenge is unknown, previous studies have shown that GMPPB catalyzes the formation of GDP-mannose, which is involved in cellular biosynthetic and post-translational modification processes [27]. A decrease or lack of GMPPB could lead to the hypoglycosylation of certain proteins, such as *N*-glycans, *O*-glycans, α-dystroglycan and glycosylphosphatidylinositol-anchors, which could disrupt cell membrane integrity and possibly macrophage function. OAS1 codes for an interferon-induced enzyme involved in the innate immune response to viruses [28], supporting reports that CIT is involved in immunomodulation.

In the OTA treatment group, the most up-regulated DEGs were adenosine monophosphate deaminase 1 (ADMP1, 10.3 fold) and CD68 (9.24 fold). ADMP1 is expressed by all cell types and is involved in driving nucleotide and energy metabolism within cells [29]. CD68 encodes a glycoprotein that is highly expressed in macrophages and functions as a scavenger receptor to clear cellular debris and promote phagocytosis [30]. Song et al. [31], however, recently used CD68 double-negative mononuclear phagocytes to show that CD68 might actually be a negative regulator of phagocytosis. The most down-regulated DEGs in the OTA treatment group were ChaC glutathione-specific gamma-glutamylcyclotransferase 1 (CHAC1, −4.27 fold) and inhibitor of DNA binding 1, dominant negative helix-loop-helix protein (ID1, −4.10 fold). In other cell types, CHAC1 is involved in oxidative stress and apoptosis in part because it promotes the degradation of glutathione [32,33]. The down-regulation of CHAC1 is in disagreement with other findings of the study herein and its role in immune cells remains unknown. Studies suggest that ID1 may have different functions under different conditions, but in general ID1 is considered a regulator of cell differentiation [34] and its inhibition herein suggests a decrease in this function. 

In the CIT + OTA treatment group, ADMP1 (9.32 fold) and fibronectin type III domain containing 7 (FDNC7, 8.71 fold) were the most up-regulated DEGs. Little information is known about the role of ADMP1 or FDNC7 in macrophages. The most down-regulated DEGs in the CIT + OTA group were ID1 (−6.72 fold) and homocysteine-inducible, endoplasmic reticulum stress-inducible, ubiquitin-like domain member 1 (HERPUD1, −5.94 fold). HERPUD1 functions as a shuttle factor transporting ubiquitinated proteins from the endoplasmic reticulum to the proteasome for recycling. Knock-down studies have shown that down-regulated HERPUD1 enhances cell susceptibility to endoplasmic reticulum stress-induced apoptosis [35]. 

Molecular and cellular functions as determined by ingenuity pathway analysis (IPA) for the DEG associated with 6 h of CIT and/or OTA exposure indicated that genes involved in cell death or cell cycle arrest were up-regulated, whereas those involved in cell cycle, cell function, and cell maintenance were down-regulated by CIT (Table 1). While previous studies have reported that CIT can induce apoptosis [36,37], our work provides evidence that CIT also can reduce cell proliferation through the inhibition of cell cycle progression. Similar to the response to CIT, genes that categorize into the cell cycle and cellular assembly biofunctions were down-regulated by OTA, suggesting cell cycle arrest and diminished cell growth, supporting findings that OTA can inhibit the proliferation of macrophages in vitro [16]. The transcript levels of several members of the cyclin, cyclin-dependent kinase and tubulin families were down-regulated by OTA, further providing molecular support for cell proliferation inhibition by OTA. Cyclins act as subunits and activate the cyclin-dependent kinases that are needed for cells to progress through the cell cycle [38]. These genes are expressed during cell cycle progression, thus a decrease in transcript levels provides evidence for the arrest of the cell cycle and the inhibition of cell proliferation. Further supporting this interpretation was a decrease in transcript-level tubulins, which leads to the disruption of the cytoskeleton and possibly cell death [39]. The stress responsive gene, growth arrest and DNA-damage-inducible protein 45 (GADD45), which is up-regulated in response to stressful growth arrest conditions, was increased in both the CIT and CIT + OTA treatment groups, but not the OTA group. 

Aside from biofunctions related to cell proliferation, OTA exposure also affected biofunctions related to gene expression and DNA replication, recombination and repair. Within these biofunctions, the function annotation DNA damage response of cells had a positive activation z-score (1.461, the z-score represents the number of standard deviations away from the mean of expression in the reference and a positive score means the value is above the mean) consistent with previous findings in other mammalian cells [40]. Several of the biological functions affected by CIT or OTA alone were also affected in the CIT + OTA group, including gene expression and cell cycle, resulting in decreased expression of members of the cyclin, cyclin-dependent kinase and tubulin families. The CIT + OTA combination also affected DNA replication, recombination and repair, potentially as a consequence of increased DNA damage and fragmentation [41]. The mycotoxins CIT and OTA have been reported to have a synergistic toxic effect on RNA transcription [24]. Our data support decreased the activation of the gene expression biofunction; however, we cannot conclusively comment on synergistic activity since interactions are likely to be affected by PM concentrations, time and biological endpoint assessment. 

The canonical molecular pathways associated with exposure to CIT, OTA or CIT + OTA at 6 h were analyzed using IPA and are presented in Table 2. Exposure to CIT increased production of ROS and increased oxidative stress levels, promoting decreased cell proliferation and increased apoptosis through induction of the caspase-signaling cascade [42]. While these cells were exposed to sublethal levels of mycotoxins and thus had low levels of cell death, the gene expression patterns were able to detect indications of cell stress and pre-apoptotic changes. In this study, pathways related to cellular and oxidative stress were affected by 6 h of exposure to CIT. These pathway changes included an increase in the transcript levels of antioxidant enzymes such as superoxide dismutase 1 (SOD1, 1.17 fold), peroxiredoxin 1 (PRDX1, 1.29 fold) and thioredoxin reductase 1 (TXNRD1, 2.72 fold), which can be activated in the presence of ROS [43]. Expression of B-cell CLL/lymphoma 2 (BCL2)-associated X protein (BAX), a pro-apoptotic protein was increased (1.15 fold) with no effect on BCL2, an apoptosis inhibitor, resulting in a slight increase in the BAX/BCL2 ratio consistent with previous findings by Kumar et al. [42]. This increase typically regulates the signaling pathways that lead to caspase activation. The current study did not show increased levels of caspase mRNA, but caspase activity, an indication of downstream regulation, was not measured and these proteins typically cluster as inactive zymogens [44].

As previously stated, OTA is thought to elicit its effects on the immune system by inhibiting immune cell proliferation and by inducing immune cell death [20,45,46]. The most-affected canonical pathways in the OTA group included the role of regulation of Igh-1b 1 (RIG1)-like receptors in antiviral innate immunity, and while there was no viral challenge in this study, the genes that were down-regulated in response to OTA in this pathway included components of the nuclear factor of kappa light polypeptide gene enhancer in B-cells 1 (NF-κB) complex-, interferon- and tumor necrosis factor (TNF) receptor-associated factors. OTA up-regulated genes associated with the activation of the eicosanoid signaling pathway, including several members of the phospholipase A2 family, which encode proteins that play a role in phospholipid remodeling, arachidonic acid release and fas-mediated apoptosis. Members of this pathway coding for subunits of each respiratory complex were up-regulated in response to OTA. In contrast, Wei et al. [47] reported that OTA decreases the function of several mitochondrial complexes and thus inhibits respiration, however these findings were obtained in response to higher rates of exposure than those of the current study. Although our findings may suggest an opposite effect, it is possible that up-regulation could provide evidence of how OTA increases cellular oxidative stress. Further research is needed to determine if increased expression of mitochondrial complexes results in altered respiration. 

When BoMacs were exposed to CIT + OTA, the most-affected canonical pathways involved the role of double-stranded RNA-dependent protein kinase (PKR) in interferon induction and antiviral response, NF-κB signaling, and transforming growth factor beta (TGF-β) signaling. Again, although there was no viral challenge, the DEG in this pathway were associated with immune system function. NF-κB signaling was inhibited and consisted of the down-regulation of several components of the NF-κB signaling pathway including several cell membrane receptors such as fibroblast growth factor receptor 1 (FGFR1, −1.25 fold), interleukin 1 receptor 1 (IL1R1, −1.53 fold), toll-like receptor 2 (TLR2, −1.14 fold) and toll-like receptor (TLR3, −1.76 fold). 

### 2.3. Transcriptional Changes after 24 h of Exposure to PM

The most up-regulated DEGs after 24 h of CIT exposure were solute carrier family 7, member 11 (SLC7A11, 9.2 fold) and the pro-apoptotic CHAC1 (6.26 fold). SLC7A11 is an amino acid solute carrier protein specific for cysteine and glutamate; its function during CIT exposure remains unknown. However, the increase in expression may be related to cellular stress levels: SLC7A11 codes for a subunit of the xCT amino acid transport system, which is the main means of increasing the production of glutathione, and is up-regulated in response to oxidative stress [48]. The most frequently down-regulated DEG after 24 h of CIT exposure were OAS1 (−4.28 fold), and tubulin beta, class 1 (TUBB, −3.13 fold). Tubulin is the major building block of microtubules. These microtubules function as the structural elements in the cytoskeleton and play an essential role in cell division, thus a decrease in expression may indicate decreases in mitosis. 

After 24 h of exposure to OTA, CD68 (22.18 fold) and histone H4 (H4, 18.92 fold) were the most up-regulated DEGs. Histone H4 regulates gene transcription, DNA replication and repair, and provides chromosome stability. The presence of extracellular H4 can impair the macrophage phagocytosis (efferocytosis) of apoptotic cells contributing to inflammation [49]. Previous work with zearalenone using epithelial cells demonstrated that members of the histone (HIST) family are down-regulated in response to mycotoxin exposure, but did not suggest a role during a mycotoxin challenge [50]. In this study, while HISTH4 and members of the HIST 1H and 2H families were up-regulated, the remaining members of the HIST family were down-regulated. The significance of this opposing response warrants further investigation and may be related to chromatin compaction, which has been reported to occur under conditions of oxygen and nutrient deprivation [51]. The most down-regulated DEG in the OTA treatment included Tensin 4 (TNS4, −14.17 fold) and CKLF-like MARVEL trans-membrane domain containing 3 (CMTM3, −5.66 fold). Tensin-4 is positively associated with proliferation and the survival of cancer cells [52], whereas CMTM3 is negatively associated with cancer cells and has a tumor-suppressive function [53].

After 24 h of exposure to CIT + OTA, similar to 6 h of exposure, AMPD1 (22.6 fold) and FDNC7 (12.1 fold) were the most up-regulated DEGs. Surprisingly, with the combination of CIT + OTA, HIST2H4A (−18.39 fold) was the most down-regulated of DEG despite being up-regulated by OTA alone. This finding is consistent with previous reports that mycotoxin exposure leads to a decrease in HIST expression [50], indicating that chromatin may undergo remodeling to allow for or prevent gene transcription, or that the histones are damaged and require replacing to keep the chromatin structure. TUBB (−12.32 fold) was also down-regulated in the CIT + OTA group. 

Overall the most-affected molecular and cellular functions after 24 h of exposure included cell death and survival, cell movement, lipid metabolism, small molecule biochemistry and cell growth and proliferation (Table 3). After 24 h of CIT exposure, gene expression patterns were similar to those of the 6-h time point, indicating increased levels of cellular and oxidative stress and decreased cell proliferation. The biofunctions most affected by OTA at 24 h were DNA replication, recombination, and repair; gene expression; RNA post-translational modification; cellular development and cell signaling. Within these biofunctions, several DNA damage inducible genes were up-regulated. These included cyclin-dependent kinase inhibitor 1A (CDKN1A, 2.32 fold), CCAAT/enhancer binding protein zeta (CEBPZ, 1.83 fold), GADD45γ, tumor protein 53 (TP53), highlighting the DNA damaging ability of OTA [54]. The gene expression biofunction included genes involved in the initiation of transcription, elongation of RNA and translation of RNA. 

While further research is needed to determine why OTA affects these pathways, one hypothesis is that these changes are the product of the cessation of cell cycle progression and the induction of DNA damage. This hypothesis was previously tested in human peripheral blood monocular cells, showing a release of ROS and an increase in markers of DNA oxidation (8-hydroxydeoxyguanosine) and DNA strand breaks [46]. When cells were exposed to CIT + OTA, the following biofunctions were affected at the 24-h time point: cell cycle, cellular growth and proliferation, gene expression, and post-translation modification. Within the cellular growth and proliferation, the functional annotation proliferation of cells and formation of cell annotations were predicted to decrease. In the cell cycle category, cell cycle progression was predicted to decrease. This prediction is supported by investigations of mitotic division, proliferation index and cell viability showing significant effects of combination of OTA and CIT incidence on hepatic cells over single exposure to the mycotoxins [55]. The gene expression biofunction had several functional annotations that were decreased: expression of RNA, transcription, and activation of DNA endogenous promoters. Within each of these biofunctions affected by CIT + OTA, individual gene expression patterns were similar to those of the 6-h time point. As part of the cell cycle biofunction, members of the cyclin, cyclin dependent kinase, and tubulin families were down-regulated in response to CIT + OTA, further supporting a decrease in cell cycle progression as demonstrated previously [16]. 

After 24 h of exposure, the most-affected canonical pathways again varied by treatment group, but overall were related to cholesterol synthesis and cell stress response (Table 4). When BoMacs were challenged with CIT, the top canonical pathways affected were all related to cholesterol synthesis. These pathways included the superpathway of cholesterol biosynthesis I, II and III. Exposure to CIT has been shown in other cell types to inhibit cholesterol synthesis [56,57]; almost all transcripts involved in the cholesterol biosynthesis pathways herein were down-regulated. These changes included a down-regulation of sterol regulatory element binding transcription factor 2 (SREBF2, −1.3 fold), considered the master regulator of cholesterol biosynthesis, and two of its cholesterol biosynthesis target genes, 24-dehydrocholesterol reductase (DHCR24, −1.23 fold) and 3-hydroxy-3-methylglutaryl-CoA synthase 1 (HMGCS1, −1.89 fold). Perhaps one of the most significant changes in this pathway was the down-regulation of 3-hydroxy-3-methylglutaryl-CoA reductase (HMGCR, −1.18 fold), the rate-limiting enzyme of cholesterol biosynthesis and target of sterol regulatory element binding transcription factor 2 (SREBF2). Similar to 6 h of exposure, the Nf-E2 related factor 2 (NRF2)-mediated oxidative stress response pathway was also predicted to increase following CIT exposure. After 24 h, within the NRF2-mediated oxidative stress pathway, there was an up-regulation of several antioxidant genes (e.g., PRDX1 (1.44 fold)), glutathione reductase (GSR, 1.78 fold), thioredoixin (TXN, 1.25 fold) and TRXRD1 (3.17 fold) and both GADD45a (1.96 fold) and GADD45g (1.79 fold), indicating cellular stress. 

After 24 h of exposure, the most-affected canonical pathways in the OTA-treated cells were P2Y-purigenic receptor signaling pathway, breast cancer regulation by stathmin1, peroxisome proliferator activated receptor (PPAR) signaling, cAMP responsive element binding protein (CREB) signaling in neurons and GADD45 signaling. The purinergic receptor (P2Y) receptor signaling on macrophages is essential for the clearance of apoptotic cells [58], therefore an inhibition of this pathway could be the result of impaired macrophage function due to OTA challenge. The activation of the PPAR signaling pathway is in contradiction to some previous work on other tissues indicating that OTA inhibits PPARγ [59], which would then suppress PPAR’s regulation of the inflammatory response. Work on astrocytes indicates that OTA increases PPARγ mRNA over time [60]. PPAR signaling is implicated in the control of inflammation, and the treatment of macrophages with PPARγ agonists can inhibit the interferon (IFN)-γ inflammatory response by specifically targeting NF-κB [61]. In the study herein, PPARγ was up-regulated (1.38 fold) by OTA exposure, which may activate PPARγ-dependent control of the inflammatory response. One possible explanation for PPARγ activation could be that initially OTA induced inflammation, which subsequently activated the PPAR pathway. However, further research is needed to test this hypothesis. GADD45 is involved in stress signaling, cell cycle control and apoptosis in the cell. In this study, GADD45g was up-regulated (2.04 fold) potentially by an increase in p53 (1.64 fold), resulting in the down-regulation of several components of this pathway involved in cell cycle progression. These changes corresponded with the up-regulation of other stress response genes such as activating transcription factor 3 (ATF3, 2.96 fold). 

After 24 h of exposure to CIT + OTA, the most-affected canonical pathways were eukaryotic translation initiation factor (EIF) 2 signaling, regulation of EIF4 and ribosomal protein S6 kinase (p70S6K) signaling, molecular mechanisms of cancer, and mechanistic target of rapamycin (mTOR) signaling. The EIF2 signaling pathway was activated in response to CIT + OTA. This pathway regulates translation initiation in response to cellular stress and is activated by pathogens [62]. While both viral and bacterial infections have been shown to activate the EIF2 signaling pathway, the study herein is the first to indicate that it can be activated by mycotoxin challenge. Since the EIF2, EIF4 and p70S6K, and mTOR signaling pathways are closely related, that the latter was decreased is unexpected. However, mTOR is also involved in the regulation of cell growth, and blocking mTOR functioning can block cell proliferation and lead to apoptosis [63]. Perhaps interesting to note is that there were few pathway and bio-functions in common between different timepoints of the same mycotoxin exposure. This could be due to changes in cellular metabolism following an acute exposure at 6 h versus a longer-term exposure at 24 h. In future studies, additional time points that fall between the two analyzed in this study may provide insight to the sequential changes that occur in cell signaling following mycotoxin exposure. 

In this study, the concentration of CIT and OTA were equivalent to 13.19 and 3.60 µg/mL. There is limited information on the in vivo exposure concentration and time-course for CIT. The concentration of OTA was within the serum concentration range reported from in vivo intravenous exposure studies [64,65,66]. In these studies, the systemic concentration of OTA between 0.1 and 4 µg/mL caused lethality to ruminants within 48 h of exposure based on in vivo studies, the time point and concentration used in this study is within a reasonable range to test acute lethal OTA toxicity. However, this concentration is much higher than blood and tissue concentrations from animals that were orally exposed to OTA-contaminated feed [67,68]. Since OTA undergoes biotransformation by rumen microbes and the liver during oral exposure to a less toxic form of ochratoxin-α, the synergism between CIT and OTA that was observed in the present study may not occur in animals unless acute lethal concentrations are reached. However, OTA may bioaccumulate in certain target tissues, so interactions at these concentrations could possibly occur in tissues such as the kidney, intestine and cutaneous fat [69,70]. It is also a possible that synergism may occur at much lower concentrations in other tissues, such as liver and embryos, that may be more sensitive to toxicological substances than the BoMacs used in this study [15,24].

## 3. Conclusions

Overall, at both time points (6 h and 24 h), exposure to CIT + OTA had a greater impact on the number of differentially-expressed genes than either CIT or OTA alone, consistent with previous studies that report an additive or synergetic effect when cells were exposed to both mycotoxins. The common gene expression patterns suggest that both CIT and OTA, and their combination, can induce oxidative stress leading to cellular stress, a slowing of cell cycle progression, and a triggering of apoptosis. Collectively, these effects can potentially inhibit the function of immune cells in exposed animals. 

## 4. Materials and Methods

### 4.1. Cell Culture and Mycotoxin Exposure 

The bovine macrophage (BoMac) cell line [71] was cultured in Roswell Park Memorial Institute 1640 medium, supplemented with 2.0 mM l-glutamine, 10% heat inactivated fetal bovine serum (FBS), 100 unit/mL of penicillin, 100 µg/mL of streptomycin, 0.25 µg/mL of amphotericin B, and 25 mM HEPES buffer. Cells were incubated at 37 °C with 5% CO_2_ in 75 cm^2^ flasks and reached at least 80% confluence before use in the study.

BoMacs (1.0 × 10^6^) were seeded into six-well, flat-bottom plates (Corning Inc., Corning, NY, USA), centrifuged for 2 min at 100 *g*, and incubated at 37 °C with 5% CO_2_. After 1 h of incubation, the cells were exposed to OTA and CIT at their respective IC_25_ (the concentration that inhibit 25% of cell proliferation, 8.91 µM and 52.72 µM, respectively), as previously determined [16]. All mycotoxins were purchased from Sigma-Aldrich (St. Louis, MO, USA) and dissolved in ethanol before further dilution with RPMI media. The IC_25_ was chosen for these PMs to provide similar stress levels in cells without causing overt cytotoxicity. Three PM treatments (OTA, CIT, or OTA + CIT) were used, as well as a solvent control (ethanol equivalent to the highest treatment concentration) for each time point. 

### 4.2. RNA Isolation and Microarray Analysis

After 6 h and 24 h of mycotoxin exposure, total RNA was extracted using the RNeasy Mini Kit according to the manufacturer’s instructions (Qiagen, Toronto, ON, Canada). RNA quantity and quality were assessed with a NanoDrop ND-1000 spectrophotometer (Thermo Scientific, NanoDrop Products, Wilmington, DE, USA) and an Agilent Bioanalyzer (Agilent Technologies, Inc., Santa Clara, CA, USA). All RNA integrity numbers (RIN) were 8.7 or above. The trials were repeated three times independently and all RNA samples were stored at −80 °C before microarray analysis. 

Labeled cRNA for all samples was made according to package directions (Affymetrix, Santa Clara, CA, USA). Labeled cRNA was hybridized to Affymetrix Bovine Gene 1.0 ST Array for 16 h at 45 °C, followed by washing, staining, and then scanning in an Affymetrix GeneChip^®^ Scanner 3000 7G. Data were normalized with robust multi-chip averaging (RMA) using the Affymetrix Expression Console software. To minimize the possibility of misleading findings, probe sets with very low signal intensity (lower than background intensity) were excluded from further analysis. The percentage of genes analysis of variance (ANOVA) expressed on the bovine array was calculated using the number of probe sets labeled present or marginal based on an applied algorithm. Subsequently, the data set was analyzed using the Affymetrix Transcriptome Analysis Console software for cluster analysis and relative gene expression.

At both time points, relative expression was obtained by comparing each mycotoxin treatment to the control using a one-way ANOVA. Genes were considered differentially-expressed relative to the control (ethanol exposure) at *p* < 0.05, irrespective of fold change. Differentially-expressed genes were uploaded into Ingenuity software (Ingenuity^®^ Systems Inc., Redwood City, CA, USA) for canonical pathway analysis. 

### 4.3. Ingenuity Pathway Analysis (IPA)

To determine the pathway, network and biological function of genes that were differentially expressed, the data set containing gene identifiers and corresponding fold changes was analyzed using Ingenuity Pathway Analysis (IPA) software (Ingenuity^®^ Systems Inc., Redwood City, CA, USA). Each identifier was mapped to its corresponding gene object in the IPA knowledgebase and overlaid onto a global molecular network developed from information in the knowledgebase.

### 4.4. Real-Time PCR 

Total RNA (0.5 μg) was reverse transcribed into cDNA using the High Capacity cDNA Reverse Transcription Kit (Applied Biosystems, Foster City, CA, USA) according to the manufacturer’s instructions. Real-time PCR was performed in triplicate using commercially available TaqMan Assay (Life Technologies) and the 7500 fast Real-Time PCR System (Applied Biosystems). The default program was used (2 min at 50 °C and 10 min at 95 °C followed by 40 cycles at 95 °C for 15 s, and at 60 °C for 60 s) with a reaction mixture volume of 20 µL in an optical 96-well plate. Glyceraldehyde-3-phosphate dehydrogenase (GAPDH) was selected as an endogenous control gene to account for any variation in the efficiency of reverse transcription and PCR. The relative quantification (RQ) was expressed as a ratio of the target gene to the control gene using the delta-delta cycle threshold method [72].

## Figures and Tables

**Table 1:** Comparison of the five bio-function-associated genes most significantly altered in bovine macrophage (BoMac) cells after 6 h of exposure to citrinin (CIT), ochratoxin A (OTA), or citrinin + ochratoxin A (CIT + OTA).

Bio-Function-Associated Genes ^1^	Number of Altered Genes
Citrinin	
Cell death and survival	339
Cellular compromise	55
Cellular function and maintenance	104
Cellular movement	56
Cell cycle	22
Ochratoxin A	
Gene expression	759
Cell cycle	254
Cellular assembly and organization	55
DNA replication, recombination and repair	166
Energy production	13
Citrinin + Ochratoxin A	
DNA replication, recombination and repair	290
Gene expression	928
Cell cycle	566
Post-translational modification	217
Cellular development	866

**^1^** The five most highly significant functions for each treatment are listed.

**Table 2:** Comparison of the five canonical pathways most significantly altered in bovine macrophage (BoMac) cells after 6 h of exposure to citrinin (CIT), ochratoxin A (OTA), or citrinin + ochratoxin A (CIT + OTA).

Most-Altered Canonical Pathways ^1^	Ratio ^2^	*p* Value
Citrinin		
NRF2-mediated oxidative stress	25/130 (0.192)	***
Endoplasmic reticulum stress pathway	7/16 (0.438)	***
Induction of apoptosis by HIV	11/51 (0.216)	***
Unfolded protein response	9/41 (0.22)	***
Glucocorticoid receptor signaling	26/204 (0.127)	***
Ochratoxin A		
Role of RIG1-like receptors in antiviral innate immunity	14/25 (0.56)	*
Eicosanoid signaling	17/34 (0.50)	*
Oxidative phosphorylation	30/78 (0.385)	*
April mediated signaling	14/31 (0.452)	*
Phospholipases	14/31 (0.452)	*
Citrinin + Ochratoxin A		
Role of PKR in interferon induction and antiviral response	20/34 (0.588)	***
NF-κB signaling	55/129 (0.426)	***
TGF-B signaling	29/60 (0.483)	**
P53 signaling	31/69 (0.449)	**
Glioma signaling	32/72 (0.444)	**

**^1^** Differentially expressed genes in CIT, OTA and CIT + OTA groups at 6 h after PM exposure underwent Core analysis, which interprets the data set in the context of biological processes, pathways and molecular networks (Ingenuity^®^ Systems); ^2^ (Differentially expressed genes)/(Total number of genes on the pathway); ^3^ A *p*-value associated with a pathway was calculated using Fisher’s exact test to determine the probability that the association between affected genes and a canonical pathway is explained by chance alone. * denotes significance at *p* < 0.05; ** denotes significance at *p* < 0.01; *** denotes significance at *p* < 0.01.

**Table 3:** Comparison of the five canonical pathways most significantly altered in bovine macrophage (BoMac) cells after 24 h of exposure to citrinin (CIT), ochratoxin A (OTA), or citrinin + ochratoxin A (CIT + OTA).

Bio-Function-Associated Genes ^1^	Number of Altered Genes
Citrinin	
Cell death and survival	814
Cellular movement	520
Lipid metabolism	229
Small molecule biochemistry	397
Cellular growth and proliferation	846
Ochratoxin A	
DNA replication, recombination and repair	272
Gene Expression	97
RNA post-transcriptional modification	109
Cellular development	72
Cell signaling	85
Citrinin + Ochratoxin A	
Cell cycle	603
Cellular growth and proliferation	1368
Gene expression	858
Post-translational modification	539
Cellular movement	762

**^1^** The five most significant functions for each treatment are listed.

**Table 4:** Comparison of the five most significant canonical pathways altered in bovine macrophage cells (BoMac) after 24 h of exposure to citrinin (CIT), ochratoxin A (OTA) or citrinin + ochratoxin A (CIT/OTA).

Most-Altered Canonical Pathways ^1^	Ratio ^2^	*p* Value ^3^
Citrinin		
Superpathway of cholesterol biosynthesis	17/23 (0.739)	***
Cholesterol biosynthesis I	8/10 (0.80)	***
Cholesterol biosynthesis II (via 24, 25-dihydrolanosterol)	8/10 (0.80)	***
Cholesterol biosynthesis III (via desmosterol)	8/10 (0.80)	***
tRNA charging	16/32 (0.5)	***
Ochratoxin A		
P2Y purigenic receptor signaling pathway	43/93	**
Breast cancer regulation by Stathmin1	58/137	**
PPAR signaling	33/69 (0.478)	**
CREB signaling in neurons	50/117 (0.427)	**
GADD45 Signaling	11/17 (0.647)	**
Citrinin + Ochratoxin A		
EIF2 signaling	85/182 (0.443)	***
Regulation of eIF4 and p70S6K signaling	57/146 (0.39)	***
Molecular mechanisms of cancer	114/365 (0.312)	***
mTOR signaling	67/188 (0.356)	***
Germ cell-Sertoli cell junction signaling	56/160 (0.35)	***

**^1^** Differentially expressed genes in CIT, OTA and CIT + OTA groups at 6 h after PM exposure underwent Core analysis, which interprets the data set in the context of biological processes, pathways and molecular networks (Ingenuity^®^ Systems); ^2^ (Differentially expressed genes)/(Total number of genes on the pathway); ^3^ A *p*-value associated with a pathway was calculated using Fisher’s exact test to determine the probability that the association between affected genes and a canonical pathway is explained by chance alone; * denotes significance at *p* < 0.05; ** denotes significance at *p* < 0.01; *** denotes significance at *p* < 0.01.

## Supplementary Materials

The following are available online at www.mdpi.com/2072-6651/9/11/366/s1, Table S1: Differentially expressed genes between bovine macrophage (BoMac) cells after 6 hours of exposure to citrinin (CIT), ochratoxin A (OTA), or citrinin + ochratoxin A (CIT + OTA) Table S2: Differentially expressed genes between bovine macrophage (BoMac) cells after 24 hours of exposure to citrinin (CIT), ochratoxin A (OTA), or citrinin + ochratoxin A (CIT + OTA), Table S3: Validation of changes in relative expression of selected genes1 in bovine macrophage (BoMac) cells exposed to citrinin (CIT), ochratoxin A (OTA) or CIT + OTA at (A) 6 h and (B) 24 h. All data have been submitted to NCBI GEO DataSets Database (Accession: GSE97329).
