# Supplementary material for: Differential Gene Expression Analysis of Bovine Macrophages after Exposure to the Penicillium Mycotoxins Citrinin and/or Ochratoxin A
Source: Toxins (Basel). 2017 Nov 13;9(11):366. doi: 10.3390/toxins9110366 (PMC5705981; doi:10.3390/toxins9110366)
Supplement: Supplementary file 1 [file toxins-09-00366-s001.zip › toxins-234919-supplementary/Toxins Abbreviations.pdf]

Abbreviations

ADMP1 adenosine monophosphate deaminase 1

ANOVA analysis of variance

ATF3 activating transcription factor 3

BAX BCL2-associated X protein

BCL2 B-cell CLL/lymphoma 2

BoMac bovine macrophage

CDKN1A cyclin-dependent kinase inhibitor 1A

CEBPZ CCAAT/enhancer binding protein zeta

CHAC1 CHAC glutathione-specific gamma-glutamylcyclotransferase 1

CIT Citrinin

CMTM3 CKLF like marvel transmembrane domain containing 3

CREB cAMP responsive element binding protein

DEG differentially expressed genes

DHCR24 24-dehydrocholesterol reductase

EIF2 eukaryotic translation intiation factor 2

EIF4 eukaryotic translation intiation factor 4

FBS fetal bovine serum

FDNC7 fibronectin type III domain containing 7

FGFR1 fibroblast growth factor receptor 1

GADD45 Growth arrest and DNA-damage-inducible protein 45

GAPDH glyceraldehyde-3-phosphate dehydrogenase

GMPPB GDP-mannose pyrophosphorylase B

GSR glutathione reductase

HERPUD1 homocysteine-inducible, endoplasmic reticulum stress-inducible, ubiquitin-like domain member 1

HIST histone

HISTH4 histone cluster H4

HMGCS1 3-hydroxy-3-methylglutaryl-CoA synthase 1

HMGCR 3-hydroxy-3-methylglutaryl-CoA reductase

IC_{25} the concentration that inhibited cell proliferation by 25%

ID1 inhibitor of DNA binding 1, dominant negative helix-loop-helix protein

IFN interferon

IL1R1 interleukin 1 receptor 1

IPA Ingenuity Pathway Analysis

mTOR mechanistic target of rapamycin

NF-κB nuclear factor of kappa light polypeptide gene enhancer in B-cells 1

NRF2 Nf-E2 related factor 2

OAS1 2'-5'-oligoadenylate synthetase 1

OSGIN1 oxidative stress induced growth inhibitor 1

OTA ochratoxin A

P70S6K ribosomal protein S6 kinase

PKR double stranded RNA-dependent protein kinase

P2Y purinergic receptor

PM *Penicillium* mycotoxin

PPAR peroxisome proliferator activated receptor

PRDX1 peroxiredoxin 1

RIG1 regulation of Igh-1b 1

RIN RNA integrity number

ROS reactive oxygen species

RQ relative quantification

SLC7A11 Solute carrier family 7, member 11

SOD1 superoxide dismustase 1

SREBF2 sterol regulatory element binding transcription factor 2

SRXN1 sulfiredoxin 1

TGF-β transforming growth factor

TLR2 toll-like receptor 2

TLR3 toll-like receptor 3

TNF tumor necrosis factor

TNS4 tensin4

TP53 tumor protein 53

TUBB tubulin beta class 1

TXN thioredoxin

TXNRD1 thioredoxin reducase 1
