# Supplementary material for: Differential Gene Expression Analysis of Bovine Macrophages after Exposure to the Penicillium Mycotoxins Citrinin and/or Ochratoxin A
Source: Toxins (Basel). 2017 Nov 13;9(11):366. doi: 10.3390/toxins9110366 (PMC5705981; doi:10.3390/toxins9110366)
Supplement: Supplementary file 1 [file toxins-09-00366-s001.zip › toxins-234919-supplementary/toxins-234919 supplementary final.pdf]

Supplementary Materials: Differential Gene Expression Analysis of Bovine Macrophages after Exposure to the *Penicillium* Mycotoxins Citrinin and/or Ochratoxin A

Kristen M. Brennan, Se-Young Oh, Alexandros Yiannikouris, Daniel E. Graugnard and Niel A. Karrow

Table S3. Validation of changes in relative expression of selected genes[1] in bovine macrophage (BoMac) cells exposed to citrinin (CIT), ochratoxin A (OTA) or CIT+OTA at (A) 6 h and (B) 24 h.

**Exposure time**		**CIT**		**OTA**		**CIT+OTA**	
	Gene	Microarray	RT-PCR	Microarray	RT-PCR	Microarray	RT-PCR
6 h	CCDN3	-1.21	-1.91	-1.45	-2.5	-1.61	-3.34
	TUBB4B	-1.81	-1.88	-1.4	-1.81	-2.05	-4.15
	SLC15A1	1.41	1.34	2.38	1.91	1.41	1.50
24h	SLC35C1	-1.46	-1.33	-4.71	-5.96	-3.1	-3.70
	SFXN3	-1.24	-1.01	-3.8	-4.06	-1.73	-1.69
	TIMP2	1.10	1.28	1.37	2.29	1.51	2.41

[1]Selected genes: CCDN3 = cyclin D; TUBB4B = tubulin beta 4B, SLC15A1 = solute carrier family 14, member A1; SLC35C1 = solute carrier family 35, member C1; SFXN3 = sideroflexin 3; TIMP2 = TIMP metallopeptidase inhibitor 2
